# Bridging the humanitarian-development divide in a protracted crisis: a case study of the use of a central plant to supply oxygen for COVID-19 case management in South Sudan

**DOI:** 10.3389/fpubh.2023.1272328

**Published:** 2023-11-10

**Authors:** Olushayo Oluseun Olu, Alex Yao Sokemawu Freeman, Joy Luba Lomole Waya, Argata Guracha Guyo, Benedict Kanu, Michael Tukuru, Sylvester Maleghemi

**Affiliations:** ^1^World Health Organization COVID-19 Preparedness and Response Team, Juba, South Sudan; ^2^African Development Bank, Monrovia, Liberia

**Keywords:** medicinal oxygen, COVID-19 case management, central oxygen production, humanitarian-development divide, protracted crises, case study, South Sudan

## Abstract

The rising demand for medicinal oxygen due to the COVID-19 pandemic exacerbated an underlying chronic shortage of the commodity in Africa. This situation is particularly dire in protracted crises where insecurity, dysfunctional health facilities, poor infrastructure and prohibitive costs hinder equitable access to the commodity. Against this backdrop, the Ministry of Health of South Sudan, with the guidance of its partners, procured and installed a pressure swing adsorption central oxygen supply plant to address the shortfall. The plant aimed to ensure a more sustainable and technologically appropriate medicinal oxygen supply system for the country and to bridge the humanitarian and development divide, which had always been challenging. This article discusses the key issues, challenges and lessons associated with the procurement and installation of this plant. The major challenges encountered during the procurement and installation of the plant were the time it took to procure and install in the face of urgent needs for medicinal oxygen and its short and long-term sustainability. Lessons learnt include the need for exhaustive and evidence-based considerations in deciding on which source of medicinal oxygen to deploy in protracted crisis settings. The successful installation and operationalization of the plant demonstrated that it is possible to bridge the humanitarian-development divide amidst the complexities of a protracted crisis and an ongoing pandemic. The Ministries of Health, with the support of its partners, should assess and document the impact of this and other similar central oxygen production plants in protracted crisis settings regarding their sustainability, cost, and effectiveness on medicinal oxygen supply. The Ministry of Health of South Sudan should expedite the finalization and operationalization of the longer-term public-private partnership and continue to monitor the quality of oxygen produced by this plant.

## Introduction

COVID-19, a disease caused by the Severe Acute Respiratory Syndrome Coronavirus 2 (SARS-CoV-2), primarily affects the upper and lower respiratory system of humans. Most people infected by the virus will manifest mild to moderate respiratory symptoms, but a small percentage of patients develop a severe illness that requires isolation, hospitalization, and medical care (1–3). It has been estimated that 20% of COVID-19 patients develop severe or critical illnesses that require oxygen therapy (4), underscoring its importance in managing the pandemic (5, 6). This has resulted in an exponential increase in the global demand for medicinal oxygen (7). This increased demand is more glaring in Lower and Middle-Income Countries (LMIC), where an estimated one million seven cubic meters of oxygen cylinders were needed for the treatment of COVID-19 as of 2021 (8). In Africa, the rising demand for medicinal oxygen exacerbated an underlying chronic shortage which is due to insufficient oxygen production capacity, as well as a lack of critical infrastructure such as reliable road networks and uninterrupted power supply, which are required for the production and distribution of the commodity (9, 10). Where medicinal oxygen is available, the price is prohibitive, another barrier to equitable access (11).

Oxygen plays a critical role in the survival of humans, animals, and plants. In the healthcare sector, it is an indispensable life-saving medicine utilized at all healthcare system levels. It is administered to treat pneumonia and COVID-19, used in the management and during the transportation of critically ill patients, and to aid asphyxiated mothers and newborns. Additionally, the commodity is used in end-of-life care within hospices and home settings (1). Medicinal oxygen differs from industrial oxygen in terms of purity and quality, as it must contain a minimum of 82% pure oxygen and be entirely free from contamination (12). Cryogenically produced oxygen should have a purity of not less than 99.5%. Oxygen purity should not be less than 93% when produced by oxygen-generation plants and between 82 and 96% when produced by oxygen concentrators (13). Therefore, in the context of this article and South Sudan, the cut-off for medicinal oxygen is 82% and above.

Naturally occurring oxygen is produced through photosynthesis by green algae and cyanobacteria in marine ecosystems and by terrestrial plants (14). Artificially, it is produced in several ways (14). The first method is through cryogenic air separation in which Nitrogen is vaporized from atmospheric air, leaving oxygen in liquid form (14). Pressure Swing Adsorption (PSA) is a second artificial oxygen production method in which dry air is passed through a molecular sieve at high pressure that adsorbs the nitrogen, leaving a gas stream of over 90% oxygen (14, 15). A third method is membrane gas separation, in which air is passed through a membrane filter, which traps slow gasses (nitrogen and argon) and releases oxygen, considered a fast gas (14). Another oxygen production method is through electrolysis, in which electricity separates water into hydrogen and oxygen (14). Medicinal oxygen is provided to patients through four main sources, namely oxygen cylinders, oxygen concentrators, central oxygen supply plants and liquid oxygen tanks (Table 1) (12). These various sources of medicinal oxygen are fraught with several challenges and opportunities in resource-poor settings. While oxygen cylinders are easiest to deploy and mostly used in such settings, they require a readily available and uninterrupted supply source and good transportation logistics from the point of production to the point of use. Oxygen concentrators are another simple-to-use source of oxygen due to their portability; however, they require an uninterrupted electricity supply to operate and local maintenance services. Central oxygen supply plants are capital-intensive and also require electricity to operate. Thus, deciding which type of oxygen supply system to use depends on several factors, such as the context, availability of infrastructure, particularly electricity at the point of production and use, cost, local capacity, and access to maintenance services and spare parts, among others (12).

**Table 1:** Description, advantages, and disadvantages of the various sources of oxygen*.

Attributes	Source of oxygen			
	Oxygen cylinder	Oxygen concentrator	Oxygen plant	Liquid oxygen
General description	Refillable oxygen vessels which are cylindrical in shape. They could be filled by a central oxygen supply plant or a liquid oxygen tank and transported to the point of use	Portable medical devices that produce oxygen from ambient air using PSA technology	Central oxygen-generating plants that use ambient air and PSA technology	Central storage point for liquid oxygen which is produced off-site and then in large tanks for redistribution to a health facility
Appropriate level of deployment	Suitable for use at all three levels of the healthcare system	Suitable for use at all three levels of the healthcare system	Secondary and tertiary	Secondary and tertiary
Cost	Moderate initial costs; high operating costs	Moderate initial cost; low operating cost	High initial cost; low to moderate operating cost	High initial cost; moderate operating cost
Maintenance requirement	Limited	Moderate	Significant maintenance by well-trained technicians	Significant maintenance by well-trained technicians
Electricity requirement	No	Yes	Yes	No
Advantages	Does not require electricity	Portable and can produce a continuous oxygen supply	Can produce oxygen continuously and cost-effective for large facilities or as a supply system for several facilities	Produces very pure oxygen and has high output
Disadvantages	Requires good transport logistics, it can be exhausted, and replenishment depends on the supplier	Requires uninterrupted power supply and produces low-pressure oxygen	High initial capital investments and requires electricity supply and adequate infrastructure to house it	Requires good transport logistics, high maintenance cost and needs adequate infrastructure

*Table adapted from the WHO-UNICEF technical specification and guidance for oxygen therapy devices – WHO medical device technical series.

Protracted crisis settings are characterized by peculiar challenges that pose significant obstacles to achieving sustainable production and supply of medicinal oxygen, particularly in Africa. These include disrupted health systems, particularly inadequate and unsustainable financing of healthcare services and inadequate human resources for health, poor infrastructure and lack of the appropriate technology to manage oxygen production. In addition to the aforementioned factors, which generally limit oxygen production and distribution capacities in Africa, insecurity stemming from armed conflicts that disrupt supply chain systems and dysfunctional health facilities that mostly lack access to electricity are features of protracted crises that limit equitable access to medicinal oxygen and other COVID-19 medical commodities. This was the situation when the COVID-19 pandemic struck South Sudan, the world’s youngest country in April 2020 (16). In this case study, we discuss the key issues and challenges associated with the sustainable production and supply of medicinal oxygen for COVID-19 case management in South Sudan. We also review the valuable lessons learnt from this experience and discuss how they could inform future efforts to address the challenge of medicinal oxygen shortage in protracted crises in Africa and globally. The lessons learnt from this case study could also be applied to implementing other public health interventions in protracted crises.

## The South Sudan context

South Sudan has been mired in decades of war, inter-ethnic conflicts, and violence, which have severely hindered the country’s socioeconomic development. Following a prolonged war with Sudan, the country gained independence in 2011, but just 2 years later, it was plunged into a civil war sparked by a political power struggle among the country’s key political leaders. This protracted conflict claimed numerous lives and displaced millions of South Sudanese into internally displaced people’s camps, exacerbating an already dire humanitarian situation. In addition to the chronic conflicts, South Sudan faces recurring natural disasters such as flooding, droughts, and disease outbreaks, compounding the humanitarian crisis in the country. These multifaceted challenges, coupled with severe micro and macroeconomic difficulties, have significantly weakened the country’s capacity for socioeconomic development. As a result, the country continues to grapple with fundamental deficiencies in basic infrastructure, including the absence of a national electricity power grid, inadequate road networks (17), limited access to safe drinking water, and a very weak healthcare system (18). According to the World Health Organization (WHO) (19), the life expectancy at birth was 58.6 years (20), while access to healthcare, maternal mortality ratio and under-five mortality rate were, respectively, 28%, 789 per 100,000 live births and 90.7 per 1,000 live births (19). As of 2020, the government health expenditure *per capita* was only US$2.63, which is grossly inadequate to provide good quality health services (21).

## The need for a central oxygen supply plant

Within this context, the country reported its first case of COVID-19 on 5 April 2020. Two months into the outbreak, the country had recorded 1,330 cases and 14 deaths with a Case Fatality Ratio (CFR) of 1.1% (22) and a cumulative of 18,368 cases and 138 deaths, with a CFR of 0.8% as of 13 September 2023 (23). As a sequel to the declaration of the COVID-19 outbreak in the country, the Ministry of Health (MoH), with the support of its partners, rapidly converted the 10-pillar emergency preparedness program into a response operation (24). Within the case management pillar, the MoH, with the support of its partners, established case management and isolation centers, trained several healthcare workers on surveillance, case management and infection control, and procured and distributed millions of US dollars’ worth of COVID-19 essential medicines, medical supplies, and equipment. Nevertheless, the management of severe COVID-19 cases was hindered by a grossly inadequate medicinal oxygen supply in the country at the initial stages of the pandemic. At that time, the country’s sole PSA oxygen plant, situated at the Juba Military Hospital, operated at a minimal capacity due to limited installed capacity and recurrent breakdowns due to poor maintenance, rendering it incapable of meeting the surging demand for oxygen.

**Figure 1:**
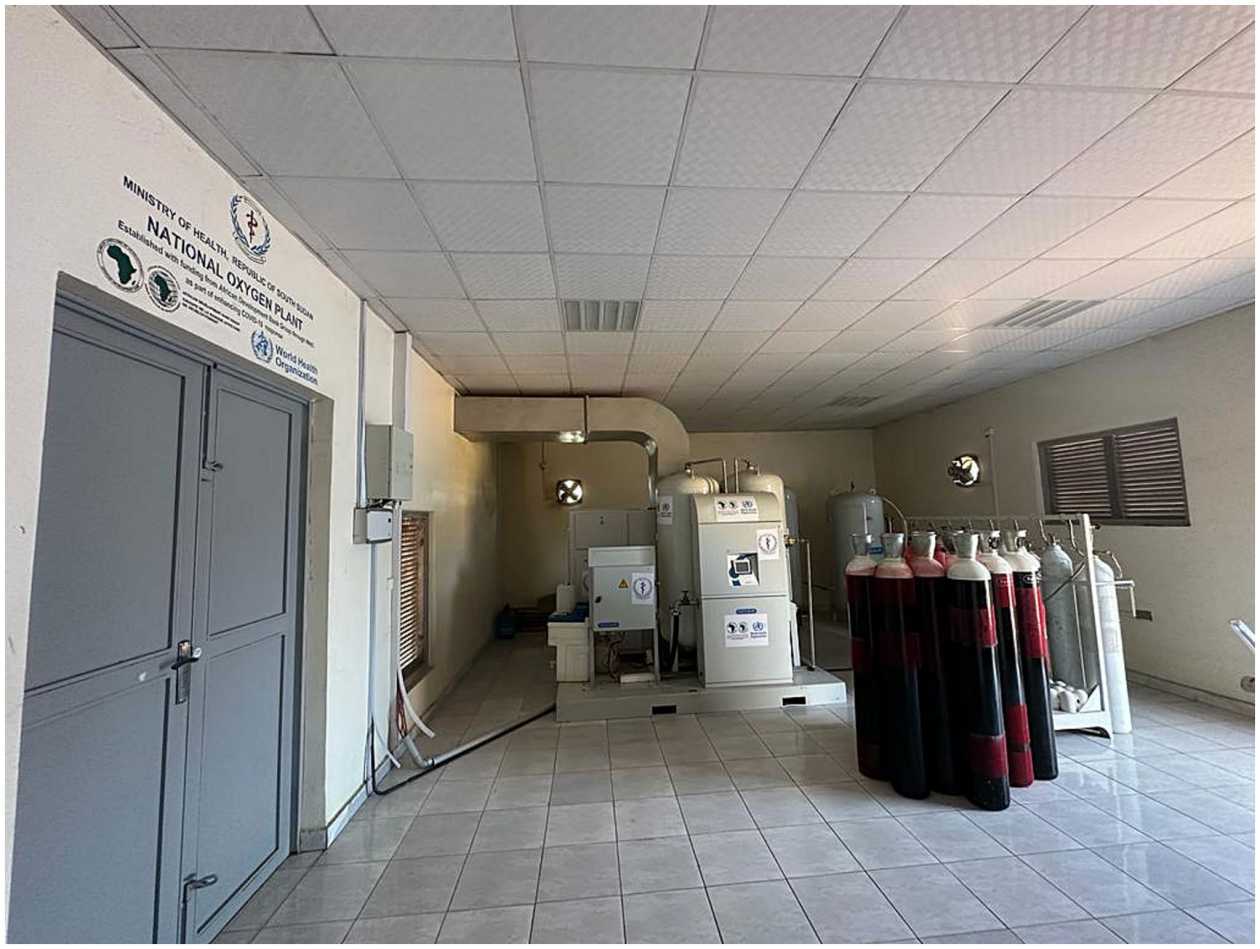
South Sudan national oxygen production plant.

The country procured cylinders of medicinal oxygen from neighboring countries to address the shortage. In addition, some health partners procured and deployed several oxygen concentrators to address the immediate oxygen needs of COVID-19 patients (25). The lack of a national power grid in the country, with all the health facilities lacking uninterrupted electricity supply, rendered most of the oxygen concentrators non-functional. Furthermore, while the oxygen concentrators were procured as a stop-gap measure to address an emergency need, their longer-term sustainability post the COVID-19 era was not guaranteed in the protracted crisis setting of South Sudan due to a lack of capacity for their maintenance at the point of use in largely remote areas. Against this backdrop, the MoH, with the guidance of the WHO and funding from the African Development Bank (AfDB), procured and installed a PSA central oxygen supply plant (Figure 1). The aims of the plant were to ensure a longer-term, more sustainable, and technologically appropriate oxygen supply system and bridge the humanitarian and development divide to strengthen the country’s health system, which had been pervasively weak. It also aimed to address the lack of power supply challenge since it was centrally installed with a reliable power supply and a backup system and produced oxygen cylinders, which could be used in remote areas without electricity or maintenance.

## Description of the plant

**Figure 2:**
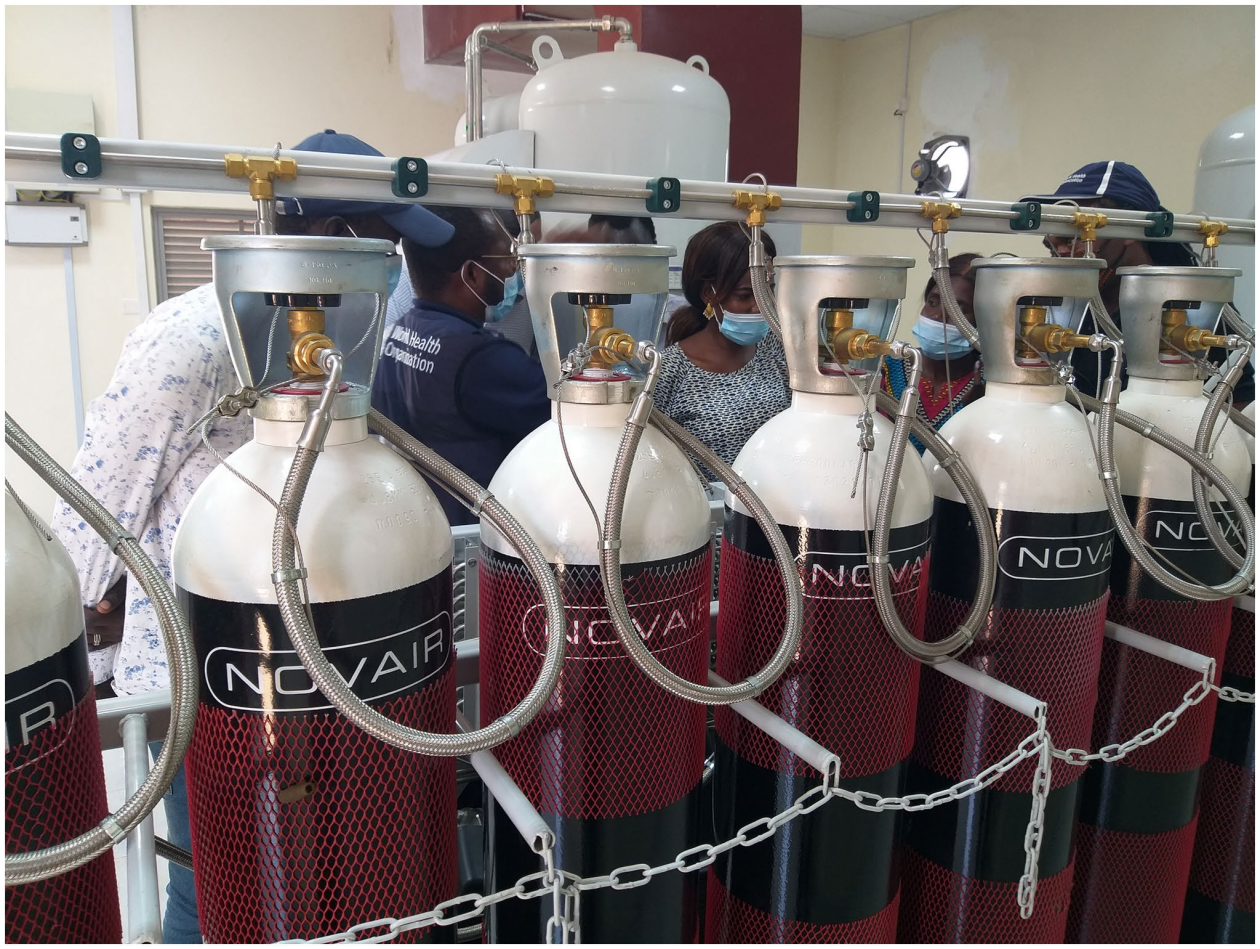
South Sudanese biomedical technicians undergoing training on the operation and maintenance of the national oxygen production plant.

The plant was manufactured by NOVAIR France and installed at the Teaching Hospital Complex in the capital city of Juba. The NOVAIR plant was selected based on the presence of the manufacturer in the sub-region (Kenya and Uganda), which guaranteed ready access to spare parts and after-sales services. Furthermore, the same type of plant had been installed in Ghana with good outcomes. It has a continuous flow rate of 29 cubic meters of above 95% pure oxygen per hour and can refill 48 oxygen cylinders, each with a capacity of 50 liters at a pressure of 200 Bar, daily. The plant was procured with essential components such as 240 spare oxygen cylinders of 50 liters each, a comprehensive four-year spare part kit, and detailed installation and user training manuals. To ensure an uninterrupted power supply, it was connected to the Juba power grid and a 150-kilovolt-ampere standby electricity power generator was installed as a backup power source. Ten South Sudanese biomedical engineers and technicians were trained on-site by NOVAIR engineers to operate, manage, and perform periodic plant maintenance (Figure 2).

## Implementation challenges

The procurement, transportation, storage, and installation of the oxygen production plant presented several challenges, potential benefits and lessons learned, which we discuss in this article.

### Long lead time

The lead time for procuring the oxygen plant was long primarily due to the lengthy administrative procedures within WHO and extensive negotiations between WHO and NOVAIR to finalize the plant’s specifications.

### Lack of dedicated technical capacity

The local biomedical engineers and technicians who were trained had other responsibilities within the MoH, thus limiting their availability to operate the plant daily. This challenge is associated with the broader fragmentation of the health system in South Sudan, where the chronic shortage of healthcare workers often means multitasking, constraining the delivery of adequate healthcare services.

### Lack of funding to cover operational costs

The MoH faced difficulties in immediately assuming full ownership and management of the plant due to its lack of funding to cover the operational, staffing, and running costs.

### Other technical difficulties

Technical challenges arose from damage to some plant components and spare parts during transportation and storage. Furthermore, the specification of some spare parts was incorrect, and some electrical installations in the purposely constructed building that housed the plant were inappropriate.

## Addressing the challenges

Creative and locally available solutions were used to address these challenges. With the support of WHO, the MoH negotiated an agreement with the African Medical and Research Foundation (AMREF), an international non-governmental organization, to support the plant’s operational, staffing, and maintenance costs while a longer-term public-private partnership model was being developed. Innovative measures were employed to locally fabricate and replace the damaged components and inadequate spare parts while waiting for replacements from NOVAIR. To overcome the challenges of transporting the oxygen cylinders, special arrangements were made with the United Nations Humanitarian Air Service to airlift the filled cylinders to the state capitals and bring back the empty ones. These collaborative efforts and creative solutions played a crucial role in ensuring the successful implementation of the project despite the challenges encountered. This aligns with the recommendations of Ross et al. who posited that expedited approaches for improving access to supplemental medicinal oxygen therapy should be implemented using locally led, context-specific and sustainable solutions (7).

## Benefits from the oxygen plant

Despite the associated challenges, the oxygen plant brought some potential benefits. Its installation in the premises of Juba Teaching Hospital, the biggest hospital in the country, which also housed the largest COVID-19 treatment center and is a short distance from the largest pediatric hospital in the country, improved access to medicinal oxygen for the treatment of COVID-19 and other diseases in the capital city and its environs. However, the plant did not have enough capacity to supply the whole country. The training of local biomedical engineers and technicians, which included didactic classroom and practical sessions (Figure 2), resulted in the transfer of technology, knowledge and empowerment of local institutions and their ability to support future expansions in the oxygen generation capacity of the country. This knowledge would be handy in procuring and installing additional oxygen plants in the country. This knowledge can also be harnessed to support other countries using South–South cooperation mechanisms. Importantly, the plant has the potential to significantly reduce the cost of oxygen in the country, as observed in similar settings (26).

## Key lessons learned

Based on the experiences in procuring and installing this central oxygen plant, we highlight six key lessons that we believe would be useful in planning, procuring, and installing oxygen plants in similar and other settings. First, deciding which source of medicinal oxygen to deploy in protracted crisis settings requires exhaustive and evidence-based considerations. While oxygen concentrators may seem to be the most appropriate for immediate deployment for emergency use in protracted crisis settings, the requirement of an uninterrupted electricity supply and maintenance facility, which is often not available in such settings, is a major constraint for the mass deployment of these devices (7). On the other hand, central oxygen supply plants, which are alternatives, also require good transport and logistic systems, which are often lacking in such settings (12). Furthermore, the likelihood of a lengthy lead time for the procurement and installation of the plant, as experienced in this case study, may be counterproductive to saving lives in an emergency. Thus, public health decision-makers should weigh the options vis-à-vis the local context and which oxygen supply strategy would be the most cost-effective and appropriate. Second, contrary to beliefs, the successful installation and operationalization of the plant demonstrated that emergency response efforts and funding could be used to build a foundation for longer-term development. Installing this plant was one of the interventions to recover and strengthen the country’s health system, which we believe would result in long-term gains. This lesson is particularly important in protracted humanitarian crisis settings like South Sudan, where the focus is always on quick emergency solutions to the detriment of early recovery and rebuilding of the health system. Third, although the plant was primarily built to support COVID-19 case management, it proved to be useful in other ways. The oxygen being produced is also used in the emergency management of other medical and surgical patients. Fourth, the plant improved accessibility to high-quality medicinal oxygen, which could be used in hard-to-reach areas without electricity or sophisticated medical equipment, thus partially resolving the oxygen shortage in the country. PSA oxygen plants are known to generally produce better quality medicinal oxygen than oxygen concentrators, and the cylinders filled and distributed by the plant could be used in any health facility with or without electricity. This can potentially improve the medical outcomes of COVID-19 patients who mostly need adequate quantity and quality of medicinal oxygen. Fifth, the successful management agreement between the MoH and AMREF ensured that the plant continued to operate in the short term and showed that public-private partnerships could work in protracted humanitarian settings. However, the long-term sustainability of the plant is still unclear and would need to be addressed by the MoH and the partners involved in its installation and operation. Furthermore, the success of this partnership will depend on both parties meeting their commitments as stated in the agreement. Sixth, the experience from this project showed that the procurement and installation of central oxygen production plants is a major undertaking, particularly in protracted crisis settings. Thus, the associated challenges, such as the long lead time for procurement, should be anticipated, and appropriate remedial actions should be planned and implemented. The benefits and lessons learnt are like those from using PSA oxygen plants in similar settings (27–29).

## Conclusion

This case study highlights the key issues, challenges and lessons associated with achieving equitable access to medicinal oxygen for managing COVID-19 and other diseases in a protracted crisis setting. It has demonstrated that exhaustive and evidence-based analyses of various sources of medicinal oxygen are required to identify the most cost-effective option in protracted crisis settings. Furthermore, it demonstrated the possibility of successfully installing and operating a central oxygen supply plant amidst the complexities of a protracted crisis like in South Sudan and the challenges of an ongoing pandemic. This is a testament to the possibility of bridging the divide between humanitarian aid and long-term development in the healthcare sector of countries in protracted crises. We believe that the lessons learnt from this, and other similar situations will stimulate the scale-up of similar initiatives to bridge the humanitarian-development divide in South Sudan and other protracted crises.

Moving forward, we propose some recommendations based on the lessons learned from this case study. First, Ministries of Health of countries in protracted crises, with the support of its partners, should assess the impact of this and other similar central oxygen plants, regarding their sustainability, cost, and effectiveness in improving medicinal oxygen supply. The lessons learnt from such an assessment should be compiled into a comprehensive compendium outlining standardized specifications, planning considerations, procurement processes, transportation and storage guidelines, installation procedures, and sustainability strategies for such plants. Second, MoH South Sudan, with the support of its partners, should expedite the finalization and operationalization of the longer-term public-private partnership. Third, MoH South Sudan should continue to monitor and document the quality of oxygen produced by this plant and consider procuring and installing additional plants in other parts of the country to supplement this plant. Fourth, WHO should conduct research studies to gain a deeper insight into how to effectively bridge the humanitarian and development divide within the health sector of countries in protracted crises. Such studies should evaluate ongoing health interventions to bridge the humanitarian and development nexus in crisis situations to identify the key issues and challenges. Such information should be used to develop comprehensive frameworks addressing the complex health system challenges these settings face.

## Data availability statement

The original contributions presented in the study are included in the article/supplementary material, further inquiries can be directed to the corresponding author.

## Author contributions

OO: Conceptualization, Project administration, Supervision, Writing – original draft. AF: Conceptualization, Project administration, Supervision, Writing – review & editing. JW: Project administration, Supervision, Writing – review & editing. AG: Project administration, Supervision, Writing – review & editing. BK: Project administration, Writing – review & editing. MT: Project administration, Supervision, Writing – review & editing. SM: Conceptualization, Project administration, Supervision, Writing – review & editing.

## References

[1] World Health Organization. (2023). Oxygen. Available at: https://www.who.int/health-topics/oxygen (Accessed May 3, 2023).

[2] Baker M Nelson S Krsnak J. Case management on the front lines of COVID-19: the importance of the individualized care plan across care settings. Prof Case Manag. (2021) 26:62–9. doi: 10.1097/NCM.0000000000000484 33507016

[3] World Health Organization. Operational considerations for case management of COVID-19 in health facility and community – interim guidance. (2020). Available at: https://www.who.int/publications/i/item/10665-331492 (Accessed May 3, 2023).

[4] World Health Organization. (2020). Oxygen sources and distribution for COVID-19 treatment centres-interim guidance. Available at: https://iris.who.int/handle/10665/331746 (Accessed September 9, 2023).

[5] Skrip L Derra K Kaboré M Noori N Gansané A. Valéa I et al clinical management and mortality among COVID-19 cases in sub-Saharan Africa: a retrospective study from Burkina Faso and simulated case analysis. Int J Infect Dis. (2020) 101:194–200. doi: 10.1016/j.ijid.2020.09.1432, PMID: 32987177 PMC7518969

[6] Kaso AW Hareru HE Kaso T Agero G. Time to recovery from Covid-19 and its associated factors among patients hospitalized to the treatment center in south Central Ethiopia. Environ Chall (Amst). (2022) 6:100428. doi: 10.1016/j.envc.2021.100428, PMID: 36632239 PMC8673952

[7] Ross M Wendel SK. Oxygen inequity in the COVID-19 pandemic and beyond. Glob Health Sci Pract. (2023) 11:e2200360. doi: 10.9745/GHSP-D-22-00360, PMID: 36853634 PMC9972372

[8] PATH. COVID-10 oxygen needs tracker. (2023). Available at: https://www.path.org/programs/market-dynamics/covid-19-oxygen-needs-tracker/ (Accessed June 2, 2023).

[9] Kitutu FE Rahman AE Graham H King C El Arifeen S Ssengooba F. Announcing the lancet Global Health Commission on medical oxygen security. Lancet Glob Health. (2022) 10:e1551–2. doi: 10.1016/S2214-109X(22)00407-7, PMID: 36162427

[10] COVID 19 and the oxygen bottleneck. Bull World Health Organ. (2020) 98:586–7. doi: 10.2471/BLT.20.020920, PMID: 33012857 PMC7463186

[11] Stein F Perry M Banda G Woolhouse M Mutapi F. Oxygen provision to fight COVID-19 in sub-Saharan Africa. BMJ Glob Health. (2020) 5:e002786. doi: 10.1136/bmjgh-2020-002786, PMID: 32532759 PMC7295423

[12] World Health Organization-United Nation Children Fund. WHO-UNICEF technical specifications and guidance for oxygen therapy devices. (2019). Available at: https://www.who.int/publications/i/item/9789241516914 (Accessed May 3, 2023).

[13] World Health Organization, Foundations of medical oxygen systems. (2023). Available at: https://www.who.int/publications/i/item/WHO-2019-nCoV-Clinical-Oxygen-2023.1 (Accessed October 16, 2023).

[14] Aljaghoub H Alasad S Alashkar A AlMallahi M Hasan R Obaideen K. Comparative analysis of various oxygen production techniques using multi-criteria decision-making methods. Int J Thermofluids. (2023) 17:100261. doi: 10.1016/j.ijft.2022.100261

[15] Emsley J. Oxygen In: Nature's building blocks: An A–Z guide to the elements. Oxford: Oxford University Press (2011). 297–304.

[16] World Health Organization. South Sudan confirms first case of COVID-19. (2021). Available at: https://www.afro.who.int/news/south-sudan-confirms-first-case-covid-19 (Accessed April 30, 2023).

[17] Ranganathan R Briceño-Garmendia C. South Sudan's infrastructure: a continental perspective. World Bank Policy Research Working Paper (5814). (2011)

[18] Jones A Howard N Legido-Quigley H. Feasibility of health systems strengthening in South Sudan: a qualitative study of international practitioner perspectives. BMJ Open. (2015) 5:e009296. doi: 10.1136/bmjopen-2015-009296, PMID: 26700280 PMC4691708

[19] World Health Organization. World health statistics (2018). Monitoring health for the SDGs, sustainable development goals. Available at: https://books.google.cg/books?hl=fr&lr=&id=sXeyDwAAQBAJ&oi=fnd&pg=PR3&ots=I53wvMiJB7&sig=60bXEgh1nRkdcQzQlgX4IheUVjg&redir_esc=y#v=onepage&q&f=false (Accessed May 1, 2023).

[20] Macharia PM Ouma PO Gogo EG Snow RW Noor AM. Spatial accessibility to basic public health services in South Sudan. Geospat Health. (2017) 12:510. doi: 10.4081/gh.2017.510 28555479 PMC5483170

[21] The World Bank. Domestic general government health expenditure per capita (current US$). Available at: https://data.worldbank.org/indicator/SH.XPD.GHED.PC.CD (Accessed September 8, 2023).

[22] Waya JLL Lako R Bunga S Chun H Mize V Ambani B. The first sixty days of COVID-19 in a humanitarian response setting: a descriptive epidemiological analysis of the outbreak in South Sudan. Pan Afr Med J. (2020) 37:384. doi: 10.11604/pamj.2020.37.384.27486, PMID: 33796197 PMC7992418

[23] World Health Organization. WHO coronavirus (COVID-19) dashboard. (2023). Available at: https://covid19.who.int/ (Accessed April 30, 2023).

[24] Reliefweb. National COVID-19 strategic preparedness and response plan June 2021 to may 2022. (2021). Available at: https://reliefweb.int/report/south-sudan/national-covid-19-strategic-preparedness-and-response-plan-june-2021-may-2022 (Accessed April 30, 2023).

[25] World Health Organization. South Sudan: WHO and UK provide oxygen concentrators to support the fight against COVID. Available at: https://www.who.int/news-room/feature-stories/detail/south-sudan-who-and-uk-provide-oxygen-concentrators-to-support-the-fight-against-covid#:~:text=The%20WHO%20and%20UK%E2%80%99s%20Department%20for%20International%20Development,severe%20shortage%20of%20medical%20oxygen%20in%20South%20Sudan (Accessed April 30, 2023).

[26] Africa Defense Forum. Somalia opens first oxygen plant during pandemic. (2021). Available at: https://adf-magazine.com/2021/10/somalia-opens-first-oxygen-plant-during-pandemic/ (Accessed June 5, 2023).

[27] World Health Organization. WHO and EU hand over life-saving medical oxygen plant to Somalia: A landmark achievement in bridging gaps in oxygen supply in the country. (2022). Available at: https://www.emro.who.int/somalia/news/who-and-eu-hand-over-life-saving-medical-oxygen-plant-to-somalia-a-landmark-achievement-in-bridging-gaps-in-oxygen-supply-in-the-country.html (Accessed June 5, 2023).

[28] China.org.cn. Oxygen producing plant built in Afghanistan's Baghlan province. (2022). Available at: http://www.china.org.cn/world/Off_the_Wire/2022-05/27/content_78240796.htm (Accessed June 5, 2023).

[29] Islamic Development Bank. New oxygen production stations are key to saving more lives from COVID-19 in Yemen. (2021). Available at: https://www.isdb.org/news/new-oxygen-production-stations-are-key-to-saving-more-lives-from-covid-19-in-yemen (Accessed June 5, 2023).

